# Nutrition-Related Mobile Apps in the China App Store: Assessment of Functionality and Quality

**DOI:** 10.2196/13261

**Published:** 2019-07-30

**Authors:** Yuan Li, Jingmin Ding, Yishan Wang, Chengyao Tang, Puhong Zhang

**Affiliations:** 1 The George Institute for Global Health at Peking University Health Science Center Beijing China; 2 Faculty of Medicine University of New South Wales Sydney Australia; 3 Public Health Graduate School of Medicine Osaka University Osaka Japan

**Keywords:** mobile phone, mobile apps, apps, nutrition, diet, food, behavior change

## Abstract

**Background:**

There are an increasing number of mobile apps that provide dietary guidance to support a healthy lifestyle and disease management. However, the characteristics of these nutrition-related apps are not well analyzed.

**Objective:**

This study aimed to evaluate the functionality and quality of nutrition-related apps in China.

**Methods:**

Mobile apps providing dietary guidance were screened in the Chinese iOS and Android app stores in November 2017, using stepwise searching criteria. The first screening consisted of extracting information from the app descriptions. Apps that (1) were free, (2) contain information on diet and nutrition, and (3) were last updated after January 1, 2016, were downloaded for further analysis. Nutritional functionalities were determined according to the Chinese Dietary Guidelines framework. Market-related functionalities were developed from previous studies and tailored to downloaded apps. The quality of apps was assessed with the user version of the Mobile App Rating Scale (uMARS).

**Results:**

Out of 628 dietary guidance apps screened, 44 were nutrition-related. Of these, guidance was provided on diet exclusively (11/44, 25%), fitness (17/44, 39%), disease management (11/44, 25%), or maternal health (5/44, 11%). Nutritional functionalities included nutritional information inquiry (40/44, 91%), nutrition education (35/44, 80%), food record (34/44, 77%), diet analysis (34/44, 77%), and personalized recipes (21/44, 48%). Dietary analysis and suggestions mainly focused on energy intake (33/44, 75%) and less on other factors such as dietary structure (10/44, 23%). Social communication functionalities were available in 42 apps (96%), user incentives were supported in 26 apps (59%), and intelligent recognition technology was available in 8 apps (18%). The median score for the quality of the 44 apps, as determined on a 5-point uMARS scale, was 3.6 (interquartile range 0.7).

**Conclusions:**

Most nutrition-related apps are developed for health management rather than for dietary guidance exclusively. Although basic principles of energy balance are used, their nutritional functionality was relatively limited and not individualized. More efforts should be made to develop nutrition-related apps with evidence-based nutritional knowledge, comprehensive and personalized dietary guidance, and innovative technology.

## Introduction

Chronic and noncommunicable diseases (NCDs) are an increasing global health challenge. According to the World Health Organization (WHO), over 14 million deaths of people between the ages of 30 and 69 years are caused by NCDs each year, of which 85% occur in developing countries [1]. Dietary risks are now the leading risk factors for NCDs, accounting for 12.2% of disability-adjusted life years (DALYs) for men and 9.0% for women [2]. Since the 1990s, WHO has called on all countries to take action to promote healthy lifestyles to prevent NCDs [3,4]. Many countries including China have developed dietary guidelines and recommendations encouraging people to keep a healthy diet in their daily life [5]. However, overall dietary patterns around the world have not improved over the past decades [6]. There remain gaps in translating knowledge into actions and achieving behavior changes toward a healthy lifestyle.

With an ever-growing telecommunications industry, mobile technology is increasingly used for health education and assistance in behavior change around the globe and in China. The number of mobile-cellular subscriptions worldwide now exceeds the global population [7]. In China, there were 772 million internet users in 2017, 97.5% of whom were mobile phone users, and almost 4 million mobile apps users [8]. People use mobile apps for games, shopping, public services, and for health counseling. There is a growing interest in mobile health (mHealth) apps for health promotion and chronic diseases prevention, mostly because of their cost effectiveness and innovations in changing behaviors [9,10]. It has been suggested that mobile phone apps have the potential to provide evidence-based health information to help people make better decisions about diet and physical activity [11]. Compared with conventional approaches, nutrition apps perform better in increasing adherence to self-monitoring [12]. These apps can also push personalized educational articles or messages to the users [13].

Although there are an increasing number of mobile apps that provide dietary guidance, their features and quality have not been thoroughly studied. The objectives of this study were to review nutrition-related apps available in China and evaluate their nutritional and market-related functionalities with the ultimate aim of identifying gaps for further improvement.

## Methods

### Search Strategy

Apps for both the iOS and Android platforms were searched, and their inclusion ot exclusion was recorded according to the Preferred Reporting Items for Systematic Reviews and Meta-Analyses flow diagram [14]. We searched iOS apps through the iOS App Store and Android apps through Tencent MyApp, the number one Android app store in China [15]. We screened apps in the following app categories: Food & Drink and Health & Fitness in the iOS App Store and Food & Takeout, Activity & Fitness, and Health & Nourishment in the Tencent Android app store. To avoid omission, we also used search keywords as a supplementation. The following Chinese search keywords, extracted from the aims of apps searched in the above categories, were used in both platforms: nutrition, diet, food, food product, dietary, catering, calorie, energy, nourishing, obesity, slimming, weight loss, weight, fitness, activity, diabetes, hypertension, hyperlipidemia, and hyperglycemia. For each category and each keyword search result, the top 100 results were included for screening, which could be regarded as the most popular apps [16]. Apps in either Chinese or English were eligible.

### Primary Analysis: Dietary Guidance App

The description and screenshots of each app were used for screening. The inclusion criteria for the primary analysis were the following: apps providing dietary advice (eg, diet plan or meal planning), food advice (eg, cooking process or characteristics of food), or nutrient information (eg, nutrient content, recommended intakes), all of which were considered to be apps providing dietary guidance. The following type of apps were excluded: (1) notebooks for dietary record without dietary analysis or nutritional advice; (2) apps for restaurant booking, take-away orders, or food sales; (3) camera apps to take pictures or videos; (4) apps designed for doctors, nurses, or nutritionists to conduct professional work; (5) educational apps for children to learn food-related words; and (6) apps providing paid offline health management service. Two pairs of reviewers screened all apps available in the iOS App Store and the Tencent Android app store on one day (November 10, 2017). The two reviewers in each group first screened independently and then reached a consensus on which apps were to be included in the primary analysis through discussion.

We extracted data on the primary purpose of each app (eg, guidance on cooking, diet, fitness, disease management, or maternal health), the developer, cost, size (MB), date of last update, number of reviews (ie, how many people rated the app), number of rating stars (0-5), number of downloads (available for Android apps only), and whether the app could be connected to peripheral devices (eg, watch or scale). For apps available on both platforms, data were extracted from the Tencent Android app store, as the iOS App Store does not display the number of downloads.

### In-Depth Analysis: Nutrition-Related Apps

Of all apps providing dietary guidance, a subsample of apps that were more closely related to nutrition, relatively integrated, informative, and accessible were selected for download in order to study their design and functionality. To identify these nutrition-related apps, the following inclusion criteria were applied: (1) contained diet, food, and nutrient information; (2) last updated after January 1, 2016; and (3) can be used free of charge. The selected nutrition-related apps were downloaded and installed on iPhones and Android phones (Huawei and Xiaomi). Apps with different names or developers but the same content were regarded as duplicates.

The 2016 Chinese Dietary Guidelines put forward the concept of a balanced diet based on the principle of energy balance. According to this, energy metabolism includes two aspects: energy intake and energy expenditure. Energy balance means keeping energy intake equivalent to energy expenditure, which is vital to maintain a healthy body weight. Dietary intake and physical activity are two important factors in energy intake and energy expenditure. Therefore, we must balance our diet and physical activity to keep a healthy body weight [5]. The Guidelines also suggested the following balanced dietary advice when providing dietary guidance: (1) “design balanced dietary recipes that follow the guidelines: keep food diversity; practice healthy cooking with recommended fat, salt, and sugar; and control energy intake to balance energy expenditure”; (2) “compare and evaluate meals by keeping rational dietary structure; that is, the intake of whole grains, deep color vegetables, milk, and beans is adequate; the energy resource from carbohydrate, fat, and protein is suitable; and the micronutrients reach the reference intakes”; and (3) “nutritional education and promotion follow the key points: promote the principle of balanced diet, encourage intake of recommended food, and suggest to eat less of some food” [5]. Accordingly, we defined five nutritional functionalities an app could provide to help follow those recommendations: (1) provide options to search for food and nutrient information; (2) record daily dietary intake using different record indicators such as weight, portion, and picture; (3) provide personalized and detailed recipes; (4) provide dietary analysis and advice, including energy-related analysis and analysis of dietary structure; and (5) push messages with nutrition-related educational materials.

We selected which technological features and market-related functionalities to study based on previous analyses and tailored this to the characteristics of our downloaded apps [17,18]. Market-related functionalities thus included social networks, interactivity, business model, intelligence technology, and connectivity to another smart device.

We recorded details of items of nutritional and market-related functionalities for each app and decided the subtotal functionalities as long as one of the detail items is positive (eg, there are four items under energy analysis functionality, including total energy intake, energy balance information, energy ratio of three meals, and energy source; if an app provided one or more of these items, it could be regarded as providing energy analysis).

### Quality Assessment of the Nutrition-Related Apps

We selected the user version of the Mobile App Rating Scale (uMARS) to assess the scientific quality of the apps [19]. uMARS is adapted for users of MARS, which has been widely used to assess apps in different fields such as mental health and cardiovascular disease [18,20-21]. uMARS presents similar key sections as those in MARS but is relatively simple to use. Although we have two reviewers doing the evaluation, they evaluated each app representing common users rather than expert developers. So we chose uMARS instead MARS to assess the apps from the user perspective and avoid understanding bias by using the simpler uMARS version. uMARS is composed of four sections: engagement, functionality, aesthetics, and information. In the engagement section, reviewers could assess whether the app was fun, interesting, customizable, interactive, or had prompts (eg, alerts, messages, reminders, feedback, enabled sharing). In the functionality section, they could assess whether the app was functional, easy to learn, easy to navigate, flowed logically, and was designed gesturally. In the aesthetic section, reviewers could score the apps according to their graphic design, overall visual appeal, color scheme, and stylistic consistency. In the information section, reviewers could assess whether the app contained high-quality information (eg, text, feedback, measure, reference) from a credible source. Overall and section-specific scores ranged from 0 to 5. During the in-depth analysis, two reviewers used uMARS to assess the apps, first independently, then reaching a consensus on the scores through discussion.

### Statistical Analyses

The number and percentage of apps with each nutritional and market-related functionality were calculated. Overall and section-specific uMARS scores were described by their maxima, minima, means, medians, and interquartile ranges. All data analyses were conducted using Stata 14.0 (StataCorp LLC).

## Results

### Search Results

**Figure 1:**
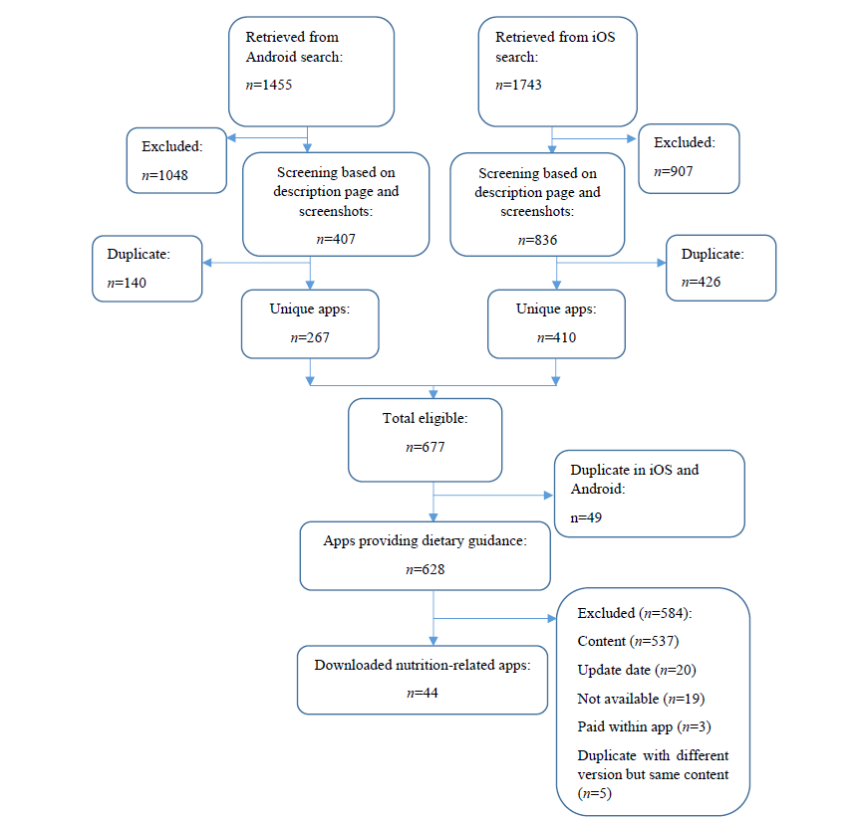
Flowchart of the selection of apps providing dietary guidance (for primary analysis) and nutrition-related apps (for in-depth analysis).

Our search found a total of 3198 apps (Figure 1). After removing duplicates, 1243 apps were screened, and 628 apps met the inclusion criteria as apps providing dietary guidance (iOS n=361; Android n=267) and were included in the primary analysis. Of these, 44 met the inclusion criteria as nutrition-related apps and were downloaded for the in-depth analysis.

### Characteristics of the Selected Apps

The majority of the 628 apps providing dietary guidance were for cooking (219/628, 34.9%), dietary guidance exclusively (114/628, 18.2%), fitness guidance (118/628, 18.8%), or disease management (95/628, 15.1%; Table 1). The apps released by individuals and corporations accounted for 42.8% (269/628) and 57.2% (359/628), respectively. User ratings with 4 and 5 scores accounted for 52.5% (330/628), while 46.8% (294/628) of the rating numbers have been rated by fewer than 100 users. Of the 267 apps with a download count available, over 50% had been downloaded more than 100,000 times (135/267, 50.1%). Most apps were last updated after January 1, 2016 (455/628, 72.5%) and were available for free (606/628, 96.5%).

Of the 44 downloaded nutrition-related apps, 41 (93%) were in the Chinese language and 42 (96%) were released by corporations; 11 (25%) were specifically aimed for dietary guidance while the rest were developed for other purposes, including fitness guidance (17/44, 39%), disease management (11/44, 25%), and maternal health (5/44, 11%). Most received a user rating of 4 or 5 stars (30/44, 68%), and of the 30 apps with a download count available, 26 (87%) had been downloaded more than 10,000 times (Table 1).

**Table 1:** Characteristics of the selected nutrition-related mobile apps.

Characteristics		All dietary guidance apps (n=628), n (%)	Downloaded nutrition-related apps (n=44), n (%)
**Aim**					
	Cooking guidance	219 (35)	—
	Dietary guidance	114 (18)	11 (25)
	Fitness guidance	118 (19)	17 (39)
	Disease management	95 (15)	11 (25)
	Maternal health	54 (9)	5 (11)
	Traditional Chinese medicine	28 (4)	—
**Platform**				
	Android	267 (43)	30 (68)
	iOS	361 (57)	14 (32)
**Developer**				
	Individual developer	269 (43)	2 (4)
	Corporation	359 (57)	42 (96)
**User rating (stars)**				
	0	2 (0)	—
	1	19 (3)	1 (2)
	2	19 (3)	2 (5)
	3	45 (7)	3 (7)
	4	169 (27)	15 (34)
	5	161 (26)	15 (34)
	Missing	213 (34)	8 (18)
**Number of ratings**				
	0-99	294 (47)	19 (43)
	100-999	110 (17)	10 (23)
	1000-9999	53 (8)	7 (16)
	≥10000	16 (3)	0 (0)
	Missing	155 (25)	8 (18)
**Download count^a^**				
	0-99	18 (7)	1 (3)
	100-999	42 (16)	1 (3)
	1000-9999	72 (27)	2 (7)
	10,000-99,999	51 (19)	9 (30)
	100,000-999,999	56 (21)	10 (33)
	≥1,000,000	28 (10)	7 (24)
**Available for free**				
	Yes	606 (96)	44 (100)
	No	22 (4)	—

^a^Download count for Android apps only (n=267 for all dietary guidance apps and n=30 for nutrition-related apps).

### Nutritional Functionalities of the Nutrition-Related Apps

In the in-depth analysis of the 44 nutrition-related apps, 91% (40/44) contained food databases to support the nutritional information provided on various foods, some of which used standard graphical displays (eg, traffic lights, stars) to show how healthy the food was. Other nutritional functionalities included nutrition education (35/44, 80%), food records (34/44, 77%), dietary analysis (34/44, 77%), and recommended recipes (21/44, 48%; Table 2). Energy was the most frequently provided nutritional information (38/44, 86%), and energy intake analysis was also the most common dietary analysis function (33/44, 75%). Only 10 out of 44 (23%) apps gave feedback and advice on dietary structure. No apps provided an analysis of protein, fat, salt, or sugar intake.

**Table 2:** Nutritional functionalities of the nutrition-related apps (n=44).

Functionalities			n (%)
**Searching food and nutrition information**			40 (91)
	**Items available in database**		
		Prepackaged food	32 (73)
		Single food	40 (91)
		Recipe	32 (73)
	**Nutritional information available in database**		
		Energy	38 (86)
		Energy-yielding nutrients	33 (75)
		Vitamins and minerals	22 (50)
		Fiber	27 (61)
	**Other information**		
		Graphical healthy rating	18 (41)
		Glycemic index	6 (14)
**Recording food intake**			34 (77)
	Quantified by weight (g)		31 (71)
	Quantified by portion		27 (61)
	Using graph to estimate amounts		9 (21)
**Providing recommended recipes**			21 (48)
	With energy requirement		19 (43)
	With specific food		21 (48)
	With amount of food		18 (41)
	With cooking guidance		12 (27)
**Dietary analysis and suggestions**			34 (77)
	**With energy analysis**		33 (75)
		Total energy	32 (73)
		Energy balance (balance intake and expenditure)	19 (43)
		Energy ratio of three meals	23 (52)
		Energy source (ratio of three macronutrients)	15 (34)
	**With dietary structure analysis**		10 (23)
		Arrangement of food groups (eg, grains, vegetables, dairy products)	6 (14)
		Estimates of micronutrients intake	7 (16)
**Nutritional education**			35 (80)
	Independent education module		26 (59)
	Intake of oil, salt, and sugar		6 (14)
	Suitable dietary structure		4 (9)

### Market-Related Functionalities of the Nutrition-Related Apps

In the in-depth analysis of the 44 nutrition-related apps, 41 (93%) enabled users to register and/or log in. Almost all apps (42/44, 96%) had at least one interaction function, such as communicating with other users or expert advisers. More than half of the apps (26/44, 59%) provided incentives such as sign-in points, badges, coupons, or rankings to improve retention of users. Eight (18%) apps offered intelligent recognition technology through identifying barcode, QR code, or photo. Eighteen (41%) apps could be connected to other health devices (eg, weighing scales, smart watches, wristbands, Bluetooth heart rate devices, blood glucose meters) (Table 3).

**Table 3:** Market-related functionalities of the nutrition-related apps (n=44).

Functionalities		n (%)
Enable user registration and/or log-in		41 (93)
**Interaction function**		42 (96)
	Blog (in app)	25 (57)
	Advice from officials and/or experts	25 (57)
	Reminder (personalized)	41 (93)
	Communicate among users	27 (61)
	Offline activity	6 (14)
	Sharing (out of app)	30 (68)
User incentive		26 (59)
Selling products		28 (64)
Advertisement		34 (77)
Crowd sourcing for data upload		2 (5)
**Intelligent recognition technology**		8 (18)
	Barcode	4 (9)
	QR code	4 (9)
	Photo	1 (2)
Peripheral applications and/or devices		18 (41)

### Quality of the Nutrition-Related Apps (uMARS Score)

**Figure 2:**
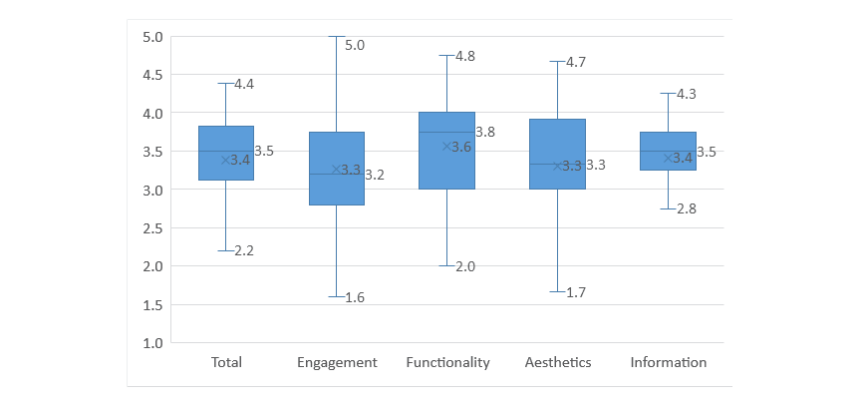
The user version of the Mobile App Rating Scale overall and section-specific scores of the nutrition-related apps (n=44). Note: The bottom and top edge of the boxes represent the first and third quartiles; the lines within the boxes represent the medians; the crosses represent the means; and the ends of the bottom and top whiskers represent the minimum and maximum values.

On the uMARS scale, nearly 80% (35/44) of the nutrition-related apps scored higher than 3 out of 5. The highest score was 4.4, with 6 (14%) apps receiving scores above 4. Most apps had an overall and a section-specific score between 3 and 4 (Figure 2). The median for the overall score was 3.5 (interquartile range [IQR] 3.1, 3.8) and the medians for the section-specific scores were as follows (in descending order): functionality (3.8), information (3.5), aesthetics (3.3), and engagement (3.2). There was also a large variety in the scores; the total uMARS score ranged from 2.2 to 4.4, uMARS engagement score ranged from 1.6 to 5.0, uMARS functionality score ranged from 2.0 to 4.8, and uMARS aesthetics score from 1.7 to 4.7. The uMARS information score had the smallest range, from 2.8 to 4.3, but its maximum 4.3 score was the lowest among the four section scores.

## Discussion

### Principal Findings

This study reviewed the features of apps providing dietary guidance and nutrition-related apps in China from nutritional and marketing perspectives. It has shown that instead of providing dietary guidance exclusively, most nutrition-related apps were developed for broader health management, including fitness guidance, disease management, and maternal health, which reflects the important role of diets in overall health [22]. It has been demonstrated that adhering to the Chinese Dietary Guidelines reduces total mortality from chronic diseases in Chinese adults [23]. The Chinese Dietary Guidelines also provide practical advice to keeping a healthy diet through recipe design, dietary analysis, and nutritional education. Dietary analysis includes the analysis of the food structure, energy sources, protein sources, nutrient intake, and use of fat and salt [5]. To review the features of the nutrition-related apps, we defined the nutritional functionalities following the principle of the Chinese Dietary Guidelines.

From the in-depth analysis, we found that the most common functionality of nutrition-related apps was the search function for food and nutrient information, while the least common was the provision of recommended recipes. This could be due to the fact that in terms of information acquisition and technology, food databases and educational functions are relatively easy to deploy in apps [24], whereas recommended recipes need to be designed by professionals. However, the reliability of the information contained in the food databases and the number of foods covered have not been assessed in this study. As the developers are primarily corporate entities, there are no authoritative organizations to participate in the development, and the accuracy of apps is worthy of verification.

In addition to providing evidence-based dietary guidance, the app design and presentation are also important to attract users [25]. Some apps used graphical forms to represent whether a food was healthy or suitable for specific groups of users or used portion sizes or reference pictures to help users record the amount food they eat. Some apps also used visual charts to display their user’s food and nutrition intake. If these kinds of visual presentations are evidence-based, they have the potential to reduce user burden and improve user experience [26]. As the graphical forms were dispersed in different functionality panels, we didn’t sum up the graphical forms of the apps and analyze their relationship with uMARS. But our informal result showed that the apps with graphical healthy rating information had higher uMARS scores (mean 3.7) than the apps without graphical healthy rating information (mean 3.2).

When the apps provided dietary analyses, the most common functionality (in 75% of the nutrition-related apps) was to calculate energy, with more than 40% of the apps using their user’s energy intake and energy expenditure to determine whether they eat too much. A likely reason for this is that the algorithm of energy balance (estimating energy intake through food and energy expenditure primarily through physical activity) is relatively easy to compute and visualize. However, a healthy diet also requires variety and dietary structure. The Dietary Guidelines for Chinese Residents recommend the consumption of at least 12 types of foods each day and more than 25 types per week. In terms of rational dietary structure, the Guidelines recommend amounts for five categories of food including cereals, animal foods, beans and nuts, vegetable and fruits, and foods only providing energy such as fats, sugars, and alcohol drinks [5]. A study has shown the positive effect of consuming a variety of foods on health [3]. However, very little information was available in the nutrition-related apps on dietary structure and the recommended intakes of different types of foods, fat, salt, and sugar. This was similar to the nutrition-related apps found in the United Kingdom [27].

In terms of app design and business operations, the nutrition-related apps provided most of the basic market functionalities, including incentives, which are important to motivate users to keep using the app [28]. In addition, several apps were able to connect to other smart devices, and a few apps supported intelligent recognition technology. With the rapid development of intelligent technology around the world, there are great opportunities for their use in nutrition-related apps.

The quality of the nutrition-related apps was considered acceptable (median overall uMARS score 3.5) with the functionality section being the best rated of the four sections assessed in uMARS (median 3.8). But the uMARS scores also showed great variety in app quality. The total uMARS score ranged from 2.2 to 4.4, and the uMARS engagement score had the widest range, from 1.6 to 5.0. According to the developers of uMARS, the score represents 1-inadequate, 2-poor, 3-acceptable, 4-good, and 5-excellent. Therefore, there were a certain proportion of apps that had low quality. In addition, for the same app there could be variety in the four section scores, which may be covered by the total score. The reason for the variety of apps is likely that there is no professional benchmark or verification for the development and release of apps in the app stores, and the quality of apps is mainly related to the ability of the developers. We found a relatively narrow range of the uMARS information score, from 2.8 to 4.3. This, to some degree, may show the homogeneity of nutrition-related information provided in the app stores. It is important to note, however, that another grading scale may be needed to more specifically assess the quality of nutrition-related information from a nutritional perspective.

### Limitations

First, our search was not exhaustive as we performed the search on only one day and only retrieved the first 100 results from each search. However, even though the apps included in our study may not be representative of the totality of apps providing dietary guidance or nutrition-related apps, the top 100 results could be regarded as the most popular apps [16]. So the functionalities reviewed here could reflect the main characteristics of the apps on the market. Second, unlike many analyses based on app markets, we did not account for download counts or user ratings (which could be used as indicators of app popularity). Our priority was to assess nutritional and market-related functionalities in order to inform future app development; therefore, all apps related to nutrition that were relatively active were included. Nevertheless, most of the nutrition-related apps we analyzed could be considered popular from a market perspective, as 85% of them have been downloaded over 10,000 times and almost 70% had a rating of 4 stars or more. Additionally, it may be inappropriate to judge the popularity of an app solely based on download counts or user ratings as both can be greatly influenced by promotional strategies of developers. In addition, download counts were not displayed in the iOS App Store. Third, even though the uMARS has been validated with 2 mHealth apps, its application to nutrition-related apps has yet to be validated, especially regarding the quality of the nutrition information.

### Conclusion

This article gave a comprehensive overview of the nutrition-related apps available on the Chinese market. Using the Chinese Dietary Guidelines as a framework to define nutritional functionalities, we found that although basic energy analysis and visual presentations were available in some apps, a limited number of apps provided comprehensive functionalities to help users adhere to dietary guidelines. On the other hand, the commercial functions of those nutrition-related apps are generally complete and some innovative technologies were attempted. The quality of the nutrition-related apps, as evaluated by uMARS, was fair but showed great variety. To improve the quality of nutrition-related apps, more effort should be made to equip them with evidence-based nutritional knowledge, comprehensive and personalized dietary guidance, and innovative technology.

## Abbreviations

DALY: disability-adjusted life year
IQR: interquartile range
NCD: noncommunicable disease
uMARS: user version of the Mobile App Rating Scale
WHO: World Health Organization
